# Genomic duplication and translocation of reactivation transactivator and bZIP-homolog genes is a conserved event in alcelaphine herpesvirus 1

**DOI:** 10.1038/srep38607

**Published:** 2016-12-07

**Authors:** Françoise Myster, Steven J. van Beurden, Océane Sorel, Nicolás M. Suárez, Alain Vanderplasschen, Andrew J. Davison, Benjamin G. Dewals

**Affiliations:** 1 Fundamental and Applied Research in Animals and Health (FARAH), Immunology-Vaccinology, Faculty of Veterinary Medicine (B43b), University of Liège, Belgium; 2 MRC - University of Glasgow Centre for Virus Research, Sir Michael Stoker Building, Glasgow G61 1QH, UK

## Abstract

Alcelaphine herpesvirus 1 (AlHV-1) is a gammaherpesvirus carried asymptomatically by wildebeest. Upon cross-species transmission, AlHV-1 induces malignant catarrhal fever (MCF), a fatal lymphoproliferative disease of ruminants, including cattle. The strain C500 has been cloned as an infectious, pathogenic bacterial artificial chromosome (BAC) that is used to study MCF. Although AlHV-1 infection can be established in cell culture, multiple passages *in vitro* cause a loss of virulence associated with rearrangements of the viral genome. Here, sequencing of the BAC clone showed that the long unique region (LUR) of the genome is nearly identical to that of the previously sequenced strain from which the BAC was derived, and identified the duplication and translocation of a region from within LUR, containing the entire coding sequences of ORF50-encoding reactivation transactivator Rta and A6-encoding bZIP protein genes. The duplicated region was further located to a position within the terminal repeat (TR) and its deletion resulted in lower ORF50 expression levels and reduced viral fitness. Finally, the presence of a similar but not identical duplication and translocation containing both genes was found in AlHV-1 strain WC11. These results indicate that selection pressure for enhanced viral fitness may drive the duplication of ORF50 and A6 in AlHV-1.

Malignant catarrhal fever (MCF) is an acute, sporadic, fatal, pan-systemic, lymphoproliferative disease of a variety of animals in the order Artiodactyla, including cattle. The main causative agents are two gammaherpesviruses grouped in the genus *Macavirus*, namely ovine herpesvirus 2 (OvHV-2) and alcelaphine herpesvirus 1 (AlHV-1). These viruses cause no apparent disease in their natural host species. Sheep are naturally infected with OvHV-2, which is responsible for the sporadic, sheep-associated form of MCF. Wildebeest are persistently infected with AlHV-1, which is the causative agent of the wildebeest-derived form of the disease^1^,^2^. The prevalence of AlHV-1 infection in wildebeest is close to 100%, and transmission to MCF-susceptible species is believed to occur mainly during the calving period and the first months of life^3^,^4^. MCF is caused by a sudden infiltration of lymphoblastic, latently infected CD8^+^ T cells into many tissues, with expression of the ORF73-encoded latency-associated protein being essential for induction of lymphoproliferative lesions^5^,^6^,^7^,^8^,^9^. The socio-economic impact of MCF has been largely underestimated^10^,^11^,^12^, implying a need for effective, low-cost vaccination strategies. The development of such strategies will depend on a firm understanding of the biology of viral infection.

Whereas OvHV-2 infection has not been established in cell culture, AlHV-1 can replicate and be maintained *in vitro.* Studies on AlHV-1 have focused mainly on two viral strains, C500 and WC11, which were isolated from an ox developing MCF and a blue wildebeest (*Connochaetes taurinus*), respectively^2^,^13^,^14^. Although the genome of strain C500 has been sequenced^15^, the genomic structure of strain WC11 has been characterized^16^ but its sequence has not. Moreover, although AlHV-1 can be maintained in cell culture, multiple passages *in vitro* have been shown to cause attenuation. In strain C500, loss of virulence has been associated with genomic rearrangements, including duplications generally involving the ORF50, A6, A7 and A10 genes^17^,^18^. A7 and A10 encode putative glycoproteins whereas ORF50 encodes a reactivation transactivator (Rta)^19^, and A6 is a positional homolog of basic leucine zipper (bZIP)-encoding genes such as those encoding Epstein-Barr virus (EBV) transcription factor Zta (also termed ZEBRA) and Kaposi’s sarcoma-associated herpesvirus (KSHV) K8 protein^20^,^21^,^22^. Although little is specifically known for AlHV-1 ORF50^23^, Rta orthologues in other gammaherpesviruses are essential for viral replication and reactivation from latency^19^, and bZIP proteins like Zta are involved in the switch needed to induce the lytic phase of the EBV life cycle in latently infected B cells^22^ while KSHV bZIP K8 is involved in the early stages of lytic DNA replication^21^. The entire genome of strain C500 has been cloned from low-passage, virulent virus as a bacterial artificial chromosome (BAC), in which infectivity and pathogenicity have been shown to be preserved^24^. The BAC clone is therefore an invaluable tool for studying the biology and pathogenesis of MCF^7^,^8^,^25^,^26^,^27^. The overall genome of strain C500 has been shown to be present in the BAC clone^24^, but the full sequence, and therefore the possible existence of undetected genetic changes, has not been determined.

In this study, we aimed to sequence the AlHV-1 BAC clone. We found that the sequence is almost identical to that of the parental strain, and we discovered and localised a duplicated and translocated region encoding ORF50 and A6 as well as partial sequences of ORF48 and A7. Since this duplication is present in a virulent BAC clone, it is not associated with a loss of pathogenicity. Indeed, we found that expression of ORF50 in the duplicated region is functional, and that it is associated with enhanced viral fitness *in vitro*. Finally, we demonstrated a similar translocation in strain WC11.

## Results and Discussion

### BAC sequencing and genome assembly

Attenuation of virulence during multiple passages *in vitro* is commonly observed with many viruses. In particular, genomic rearrangements have been observed in several laboratory strains of herpesviruses^28^,^29^, and rearrangements of the AlHV-1 genome during multiple passages in cell culture have been described^16^,^17^,^18^. Some of these rearrangements, including duplications and translocations, have been associated with the production of increased numbers of cell-free viral particles and loss of pathogenicity. A decade ago, we cloned an infectious, pathogenic form of the strain C500 genome as a BAC^24^, which is now used extensively in studies of MCF pathogenesis. To characterize this clone more fully, we determined its sequence and also that of derived virus. Virus derived from BAC was reconstituted by transfection of the BAC clone into MacT-Cre cells and consequent Cre-mediated excision of the BAC vector, which is located in the viral microRNA-rich region between ORF11 and ORF17^30^. The respective numbers of sequence reads obtained were 1,344,976 and 1,586,106, of which 99 and 22% matched the final sequences with average coverage values of 1,366 and 379 reads per nucleotide.

### BAC sequence analysis and comparison with strain C500

The AlHV-1 genome consists of a long unique region (LUR) flanked at each end by multiple copies of a terminal repeat (TR)^15^. The sizes of LUR in the BAC clone and the reconstituted viral genome were 140,575 bp and 130,815 bp, respectively, the sole difference being the presence of the BAC vector in the former. The sequence of ORF73, which has a high G + C content and contains several reiterations, was confirmed in its entirety in the BAC clone by high-fidelity PCR followed by Sanger sequencing. The sequence of LUR is very similar to that of the reference sequence for strain C500 (GenBank accession AF005370)^15^ (Table 1). TR is 1,108 bp in size, and, on the basis of coverage values, is present in multiple copies in both the BAC clone and reconstituted viral DNA. Its sequence differs from those determined previously for strain C500 (GenBank accessions AF005363-AF005368)^15^ only in the lengths of two variable reiterations and by a single nucleotide substitution. These results confirmed that the BAC clone contains the complete AlHV-1 genome and further validated its use as a tool for studying the pathogenesis of MCF.

### Genomic duplication and translocation in strain C500

**Figure 1:**
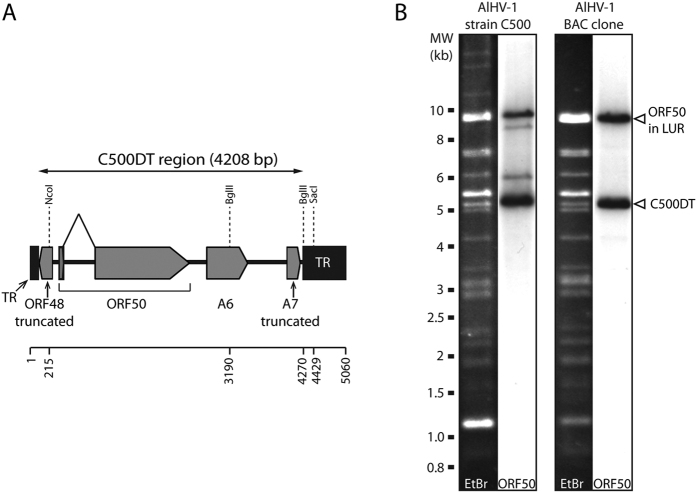
Presence of a duplicated region in parental strain C500 and the BAC clone. (**A**) Diagram of C500DT showing the duplicated region from LUR containing a part of ORF48, ORF50 and A6 in their entirety and a part of A7, inserted into a copy of TR. Nucleotide positions are indicated. (**B**) Southern blot of SacI-digested DNA showing the presence of the duplicated region in the BAC clone and the parental virus. The ORF50 probe was produced by PCR using primers C500-1 and C500-2^24^. EtBr indicates ethidium bromide-stained lanes prior to blotting. The entire lanes analysed by Southern blotting are shown.

The sequence of the BAC clone implied the presence of a 5,060 bp region consisting of a TR unit in which 256 bp had been replaced by a duplicated translocation of a 4,208 bp region from LUR (nucleotides 72,499–76,706 in the reference sequence; termed C500DT) containing the first 163 bp of ORF48 coding sequence, complete ORF50, complete A6 and the first 136 bp of A7 coding sequence (Fig. 1A). Interestingly, the duplicated region had complete sequence homology with the sequence present in the LUR. This duplication is similar to one of several genomic rearrangements described previously after passages of the original, low passage strain C500 in cell culture^17^,^18^. Digestion of the BAC clone and the original strain with SacI, which cuts TR once but not C500DT, followed by Southern blotting using an ORF50 probe, led to the detection of a ~5 kbp band comprising C500DT in both (Fig. 1B). It seemed likely that C500DT is located among the multiple TR copies in the BAC clone, but its precise position was not apparent from the sequence data or this experiment. Additional fainter bands were detected by Southern blotting of strain C500, which could reveal additional genomic rearrangements involving ORF50 that have previously been observed^18^.

**Figure 2:**
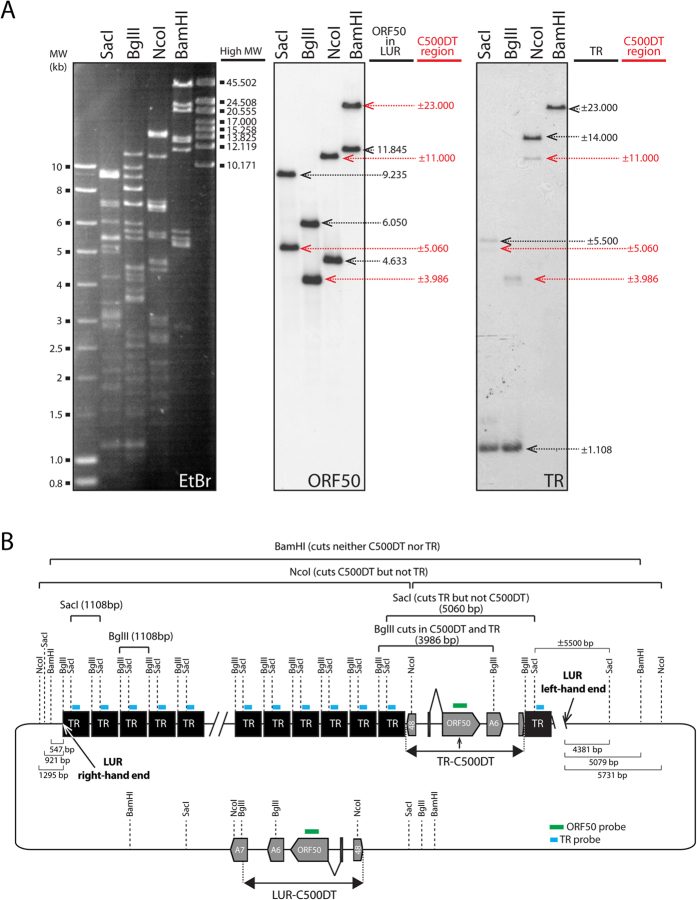
Localization of the duplicated region in the BAC clone by Southern blotting. (**A**) Southern blot of the BAC clone digested with various restriction endonucleases and hybridized with ORF50 or TR probes. The sizes (anticipated and deduced) of bands from LUR-C500DT or TR-C500DT are shown in black and red, respectively. The ORF50 probe was produced by PCR using primers C500-1 and C500-2^24^. The TR probe was produced after AvrII/SmaI digestion of plasmid pBluescribe-C500RE BS^15^ and purification of the band corresponding to a 235 bp sequence of TR (nt 490–724 of TR). EtBr indicates ethidium bromide-stained lanes prior to blotting. Entire gels and blots are shown with ladders of medium (MW) and high molecular weight (High MW) standards. (**B**) Diagram of the BAC clone showing the positions of restriction sites used to locate the duplicated region. Locations of the ORF50 and TR probes used in A are shown by green and blue lines, respectively.

**Figure 3:**
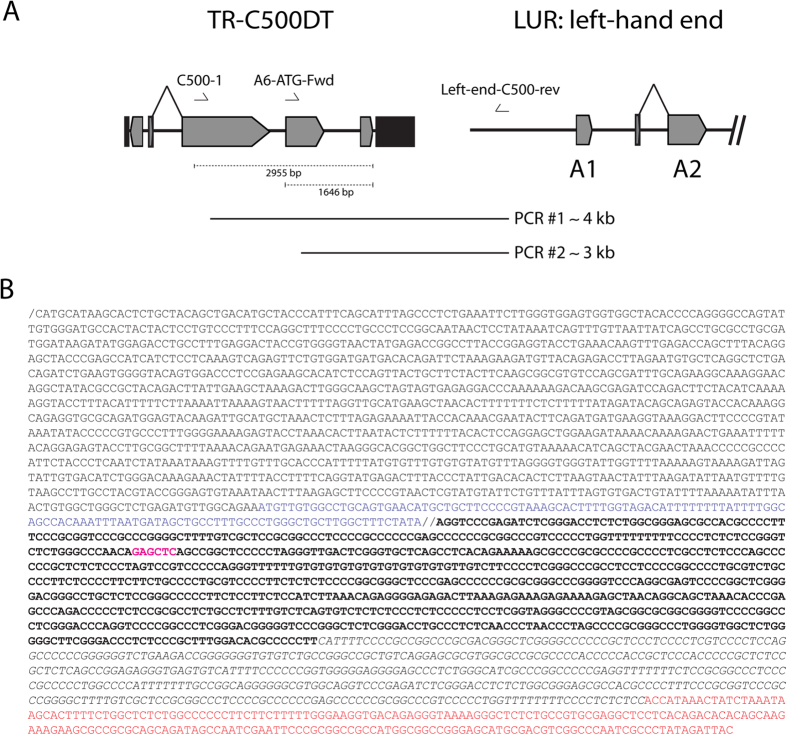
Precise localization of the duplicated region in the BAC clone by PCR and Sanger sequencing. (**A**) Locations of primers used. (**B**) Sanger sequence of the relevant parts of PCR #2. The left end of LUR is shown in red, and double-lined breaks indicate the junction between C500DT and TR. The truncated A7 sequence in TR-C500DT is shown in blue. The sequence in bold indicates the last 800 bp of TR. The italicized sequence indicates the first 452 bp of TR. Purple sequence indicates SacI restriction site.

To localize the duplication, we carried out Southern blotting of the BAC clone digested with a range of restriction endonucleases and probed using ORF50 and TR sequences (Fig. 2). In this experiment, we denoted the original sequence as LUR-C500DT and the duplication as TR-C500DT (Fig. 2A). SacI, which cuts TR but not C500DT, generated a ~5 kbp fragment to which the ORF50 probe hybridized. The TR probe hybridized to a hypermolar signal at ~1 kbp that was consistent with the 1108 bp TR sequence detected in the sequencing experiment, in addition to a ~5.5 kbp fragment which could correspond to the left-hand end TR-LUR junction and a ~5 kbp fragment that could include C500DT (Fig. 2B). BglII, which cuts both TR and C500DT, produced a ~4 kbp fragment detected by both probes. BamHI cuts neither sequence, and both probes detected a ~23 kbp fragment that contained the TR array, TR-C500DT, and the left (5,079 bp) and right (547 bp) regions of LUR. These findings indicate that the TR array occupies ~12.3 kbp and contains ~11 copies of TR. From these observations, we could reconstruct the BAC sequence as a circle, making the sequence “complete” (Fig. 2B). NcoI cuts C500DT but not TR, and both probes detected an ~11 kbp fragment, the weaker signal from the TR probe suggesting that this fragment contained relatively few TR units, and therefore that TR-C500DT may be localised near one end of the TR array and close to one end of LUR. Based on the locations of the NcoI sites, we hypothesized that the duplicated region was located near the left end of LUR. We designed primers in TR-C500DT, specifically in ORF50 (C500-1) and A6 (A6-ATG-Fwd), and near the left end of LUR (Left-end-C500-rev). The results of amplifying, cloning and Sanger sequencing one of the PCR products (PCR #2 in Fig. 3) demonstrated that C500DT was duplicated within a TR unit and separated from the left end of LUR by the first 452 bp of a TR unit (Fig. 3 and Table 2).

### Characterization of a strain C500 mutant lacking TR-C500DT

**Figure 4:**
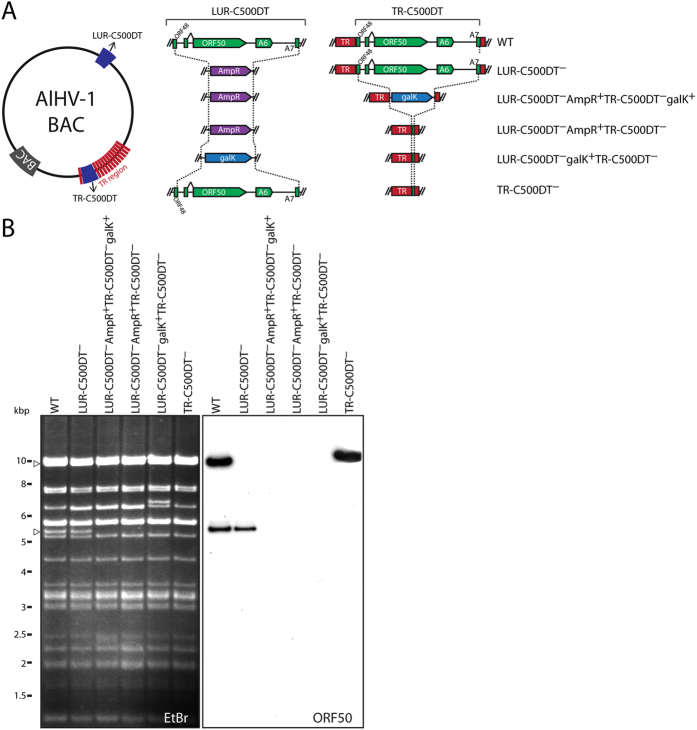
Deletion of TR-C500DT. (**A**) Outline of the recombineering strategy used to remove C500DT from TR and generate the TR-C500DT^−^ recombinant strain. (**B**) Southern blotting of the constructs generated. The ORF50 probe was produced by PCR using primers C500-1 and C500-2^24^. EtBr indicates ethidium bromide-stained lanes prior to blotting. The entire gel and blot analysed by Southern blotting are displayed.

The duplicated region in the BAC contains the entire ORF50 and A6 genes, which encode putative homologues of gammaherpesvirus Rta and bZIP proteins, respectively. These proteins are involved in the regulation of gammaherpesvirus lytic *versus* latent infection^19^,^20^,^22^. We hypothesized that the presence of more than one functional copy of these genes might improve viral fitness and be selected during viral replication *in vitro*. We addressed this hypothesis initially by deleting TR-C500DT from the BAC (Fig. 4A) and characterizing the structure of the resulting BAC by Southern blotting (Fig. 4B). LUR-C500DT was removed by inserting a β-lactamase gene (AmpR, LUR-C500DT^−^), and then TR-C500DT was removed by inserting galK (LUR-C500DT^−^AmpR^+^TR-C500DT^−^galK^+^). To generate a cleaner deletion of TR-C500DT, galK was counter-selected in order to replace it by a 100 bp sequence corresponding to the left and right ends (50 bp each) of C500DT (LUR-C500DT AmpR^+^TR-C500DT^−^). Then, LUR-C500DT was reverted by replacing AmpR with galK (LUR-C500DT^−^galK^+^TR-C500DT^−^) and counter-selecting with a PCR-generated LUR-C500DT sequence (TR-C500DT^−^). Thus, in the strain TR-C500DT^−^, the TR-C500DT entire region was replaced by the two flanking 50 bp sequences of the C500-DT region, corresponding to sequences present in ORF48 and A7. The resulting constructs were verified by restriction endonuclease digestion and Southern blotting (Fig. 4B), and the integrity of LUR-C500DT in TR-C500DT^−^ was verified by Sanger sequencing of the entire region.

**Figure 5:**
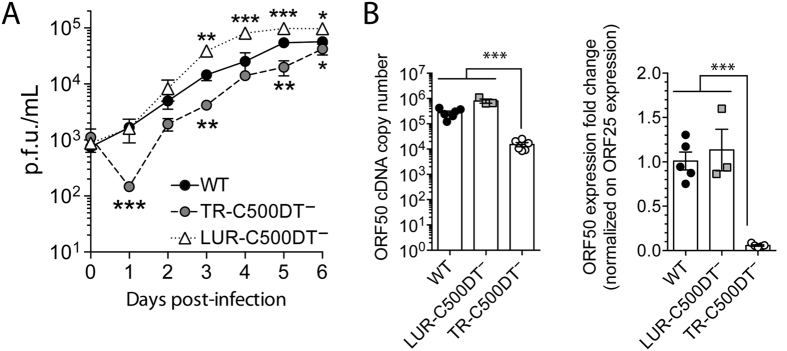
Effects of the duplicated region on viral fitness *in vitro*. (**A**) Multistep growth curves of WT, LUR-C500DT^−^ and TR-C500DT^−^ viruses in BT cells. Data are means ± S.D. from triplicate independent measurements and representative of two independent experiments with similar results. Statistical analysis was done by two-way ANOVA with the Bonferroni test for multiple comparisons; *p ≤ 0.01, **p ≤ 0.05, ***p ≤ 0.001. (**B**) ORF50 mRNA expression in MDBK cells 3 days post-infection (moi = 0.01) with WT, LUR-C500DT^−^ or TR-C500DT^−^ viruses. Expression levels are given as absolute copy numbers from an RT reaction of 1 μg of cytoplasmic RNA and as normalized ORF50/ORF25 ratios. Data are means ± S.D. from measurements carried out in triplicate. Statistical analysis was done by Student *t* test, ***p ≤ 0.001.

MacT-Cre cells were transfected with the wild-type (WT), LUR-C500DT^−^, LUR-C500DT^−^galK^+^TR-C500DT^−^ and TR-C500DT^−^ BACs, all of which generated progeny except double-deleted LUR-C500DT^−^galK^+^TR-C500DT^−^, consistent with previous studies demonstrating the essentiality of the Rta homologs for viral replication. Successful growth of the LUR-C500DT^−^ virus (Fig. 5A) strongly suggested that ORF50 in TR-C500DT is functional. It was interesting to notice that the LUR-C500DT^−^ virus displayed increased growth *in vitro* whereas containing only one copy of the ORF50 and A6 in the TR. Importantly, removal of C500-DT from the LUR not only resulted in the absence from this region of ORF50 and A6, but also of the first 163 bp of ORF48 and the first 136 bp of A7. Thus, the expression of these genes are likely altered and could explain the growth difference. The growth kinetics of the TR-C500DT^−^ virus in cell culture were then investigated. The absence of TR-C500DT resulted in reduced viral growth compared to WT (Fig. 5A).

We then determined whether ORF50 was expressed differentially after infection with the WT, LUR-C500DT^−^ or TR-C500DT^−^ viruses. RT-qPCR analyses (Fig. 5B) revealed a significantly lower expression of ORF50 in cells infected with TR-C500DT^−^ compared to WT while ORF50 expression from LUR-C500DT^−^ resulted in expression levels slightly higher to the WT virus. The ORF50 mRNA expression levels can be dependent on the replication efficiency and not only on the number of active gene copies present in the genome. Thus, to further examine the effect of the duplication on mRNA expression levels, the copy numbers of ORF50 mRNA were normalized to those of ORF25 mRNA encoding the major capsid protein. Normalized ORF50 mRNA expression levels were significantly lower in TR-C500DT^−^ infected cells, indicating that downregulated expression of ORF50 was not due only to reduced levels of infection. Interestingly, normalized ORF50 mRNA levels of the LUR-C500DT^−^ virus were not significantly different from the parental WT strain. This observation was surprising and could suggest that the TR region regulates gene expression, leading to heightened levels of mRNA synthesis of ORF50. In this case, the majority of ORF50 gene expression in the WT virus might result from expression of the ORF50 gene present in the TR-C500DT region.

### Strain WC11 also contains a duplication and translocation of ORF50 and A6

**Figure 6:**
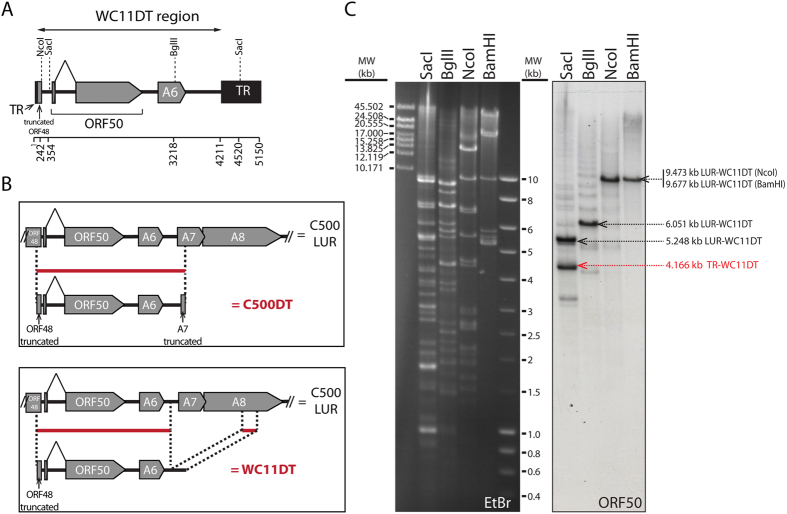
ORF50 and A6 are duplicated in strain WC11. (**A**) Diagram of the duplicated region (WC11DT) containing ORF50 and A6 identified by sequencing. (**B**) Diagrams of the aligned C500DT and WC11DT sequences on the C500 LUR sequence. Red lines correspond to the duplicated regions from LUR. (**C**) Localization of the duplicated region by endonuclease restriction and Southern blotting. The ORF50 probe was produced by PCR using primers C500-1 and C500-2^24^. Red font indicates the duplicated fragment containing ORF50. Black font indicates the fragment containing ORF50 in LUR. EtBr indicates ethidium bromide-stained lanes prior to blotting. Entire gels and blots are shown with ladders of medium (MW) and high molecular weight (High MW) standards.

In the experiments described above, we observed that both ORF50 and A6 are duplicated in the original strain C500 and the BAC clone derived from it. Since duplication of ORF50 has also been described in other gammaherpesviruses, such as bovine herpesvirus 4 (BoHV-4)^31^ and may have been selected *in vitro*, we investigated whether similar events may have occurred in AlHV-1 strain WC11, which has been passaged many times in cell culture after isolation from a wildebeest calf^16^,^32^. Sequencing of this strain from another study revealed the presence of a duplicated translocation that is very similar to that in the C500 BAC clone. Thus, we identified a 4,177 bp sequence flanked by TR sequences and containing the complete coding sequences of ORF50 and A6 (termed WC11DT in Fig. 6A). This observation was surprising and could suggest either convergent evolution or a common phylogenetic origin of both strains. Convergent evolution would suggest that the duplication of the genomic region containing ORF50 and A6 has occurred in both strains independently, either in the infected host *in vivo* or during passages in cell culture. However, despite being independent isolates^2^,^13^, the strains WC11 and C500 might share a common ancestor in which the duplication has occurred. We further aligned the sequence of C500DT and WC11DT (Supplementary Fig. S1). Although both sequences were highly conserved with only 4 single nucleotide polymorphisms, the last 501 and 421 bp of C500DT and WC11DT, respectively, were dissimilar. Further sequence analysis identified that the last 421 bp of WC11DT were a duplication of part of A8 sequence, which was absent in C500DT (Fig. 6B and Supplementary Fig. S2). Such dissimilar structures of the C500DT and WC11DT rather suggests that despite being phylogenetically very close, strains C500 and WC11 have acquired the duplicated region during independent recombination events. Next, an experiment involving restriction endonuclease digestion of WC11 DNA purified from viral particles and Southern blotting using an ORF50 probe was carried out (Fig. 6C). SacI cuts LUR and also has a single restriction site in one of the two TR variants in this strain, and BglII, NcoI and BamHI cut LUR but not TR. As the viral genome in virions is linear with a random distribution of multiple TR units at each end of LUR, the use of endonucleases that cut LUR but not TR enabled the detection by the probe of LUR-WC11DT but not TR-WC11DT, thus suggesting that TR-WC11DT is present in the TR array (laddering signal observed in the corresponding lanes). The detection of two bands by the probe after digestion with SacI validated the 4,177 bp duplication observed by sequencing.

In the present study, we sequenced the genome of the AlHV-1 strain C500 BAC clone and identified a duplicated region of LUR that had been translocated into a TR unit close to the left end of LUR. The duplicated region was also present in the genome of the parental virus, and contained ORF50 and A6, two genes involved in promoting lytic viral replication in other gammaherpesviruses^21^. Interestingly, duplication of these genes was not restricted to the BAC clone of strain C500, as a very similar but not identical duplication was identified in strain WC11. It is likely that AlHV-1 ORF50 and A6 function similarly to their orthologues in other gammaherpesviruses. Whereas the absence of the duplicated region impaired virus growth, supporting the essentiality of ORF50, specific deletion of C500DT reduced viral fitness. Thus, the expression of ORF50 and A6 might enhance viral growth in cell culture, so that a selection pressure for their duplication might drive genomic rearrangements either *in vivo* or during passage in cell culture. Interestingly, OvHV-2 growth *in vitro* has never been established^33^. It would be interesting to investigate the presence or not of a similar duplication of ORF50 and A6 homologs in the genome of OvHV-2. Although such rearrangements *in vitro* can potentially involve gene sequences that are essential for virulence *in vivo* as observed in the high passage, attenuated strain C500^18^, low passage virus reconstituted from the BAC clone (containing the duplicated C500DT region) maintains its ability to induce MCF in susceptible species^24^, suggesting that the genomic rearrangement observed in the BAC clone has no major impact on MCF pathogenesis. In conclusion, the duplication and translocation of ORF50 and A6 may result from selection to improve viral fitness. It would be interesting to investigate whether the duplication has strictly occurred during passages in cell culture and therefore characterized as an artefact of culture or whether it has occurred during coevolution of AlHV-1 with its natural host, wildebeest. In which case, the duplication of ORF50 and A6 should be observed in virus samples from wildebeest or naturally-infected MCF-susceptible species.

## Materials and Methods

### Cell lines and viral strains

Bovine turbinate fibroblasts (BT, ATCC CRL-1390) and MacT-Cre cells^26^,^34^ were cultured in Dulbecco’s modified essential medium (D-MEM, Invitrogen Corporation). Madin-Darby bovine kidney cells (MDBK, ATCC CCL-22) were cultured in modified essential medium (MEM). All cells were cultured in presence of 10% (v/v) foetal calf serum (FCS) (BioWhittaker). The original AlHV-1 strain C500^13^ (low passage) and strain WC11^14^ were provided generously by Prof. D. M. Haig, University of Nottingham. Viruses were generated from AlHV-1 BAC clones in MacT-Cre cells and then grown in BT cells^24^. The original strain C500 and the reconstituted viruses were maintained for <5 passages in BT cells.

### Next-generation sequencing

BAC clone DNA^24^ was purified by using the Large Construct DNA prep kit (Qiagen). Viruses were generated from AlHV-1 strain C500 BAC clones in MacT-Cre cells, grown in BT cells, and purified from the supernatant by ultracentrifugation (100,000 × *g*) through a 30% (w/v) sucrose cushion for 2 h at 4 °C. DNA was extracted from the pelleted virus^35^. Paired-ended, 150 nucleotide sequence reads were generated for the strain C500 BAC clone and the reconstituted virus by using a MiSeq DNA sequencer running version 2 chemistry (Illumina) and assembled by using methods described previously^36^. The sequence of the TR region of strain WC11 was derived as part of a similar project.

### Nucleotide sequence accession numbers

The sequences of the strain C500 BAC clone and the reconstituted virus were deposited in GenBank under accession numbers KX905134 and KX905135, respectively. The sequence of the TR region of strain WC11 was deposited in GenBank under accession number KX905136.

### Comparative genomics analysis

Sequence alignments were performed by using MAFFT on “auto strategy” settings (http://mafft.cbrc.jp/alignment/server/) and further analysed for sequence divergence by using Jalview (http://www.jalview.org). Nucleotide polymorphism and synonymous vs nonsynonymous mutations in protein coding sequences were identified (Table 1).

### Production of recombinant AlHV-1 C500 BAC viruses

To remove the TR-C500DT region from the BAC clone, multistep, sequential approaches were taken in *Eschericia coli* strain SW102 bacteria (Fig. 4)^37^. First, LUR-C500DT was replaced by a β-lactamase (ampR) gene via an PCR amplicon produced using primers LUR-C500DT-AmpR-Fwd and -Rev (Table 2) and pcDNA3.1 as DNA template. TR-C500DT was then replaced by galK via a PCR amplicon produced using TR-C500DT-galK-Fwd and -Rev, and pgalK as template. Next, the galK cassette in TR was removed by using annealed oligos C500DT-clean-Fwd and -Rev. Finally, reversion of LUR-C500DT was performed via a PCR fragment produced using primers LUR-C500DT-galK-Fwd and -Rev and pgalK as template, followed by counter-selection for recombination via a PCR fragment produced by primers LUR-C500DT-Fwd and -Rev and BAC DNA as template, to generate the TR-C500DT^−^ BAC plasmid. Infectious viruses from generated recombinant BAC clones were reconstituted by transfection in MacT-Cre cells to excise the BAC cassette, and propagated in BT cells to generate the corresponding BAC-excised strain, as described previously^24^.

### Southern blotting

DNA was digested with restriction endonucleases, separated on 0.7% (w/v) agarose gels with ladders of medium-sized (SmartLadder, Eurogentec) and high molecular weights (GeneRuler High Range DNA Ladder, Thermo Fisher) standards. Gels were then transferred to Amersham Hybond-XL blotting membranes (GE Healthcare) by capillary transfer^38^. DNA fragments used as probes for hybridization were labelled with α-[^32^P]dCTP (specific activity, 3000 Ci/mmol; Perkin Elmer) using a random-primed DNA labelling kit (Roche). Membranes were hybridized at 65 °C for 18 h, washed, and exposed to Amersham Hyperfilm MP (GE Healthcare).

### Multistep growth curves

*In vitro* growth kinetics of TR-C500DT^−^ and LUR-C500DT^−^ viruses were compared to those of WT by using a published protocol^8^.

### RT-qPCR

Cytoplamic RNA was extracted from AlHV-1-infected MDBK cells (RNeasy mini kit, on-column DNAse I treatment, Qiagen), and cDNA was synthesized from 1 μg of RNA by using Superscript III Reverse Transcriptase (Thermofisher). Transcription of ORF50 and ORF25 was quantified as described previously^7^, using iQ-Sybr Green reaction mix and a CFX96 Touch Real-Time PCR Detection System with CFX Manager v3 software (Bio-Rad).

### Statistical analyses

Statistical analyses were conducted by using GraphPad Prism v6 software.

## Additional Information

**Accession codes:** The sequences of the strain C500 BAC clone and the reconstituted virus were deposited in GenBank under accession numbers KX905134 and KX905135.

**How to cite this article**: Myster, F. *et al*. Genomic duplication and translocation of reactivation transactivator and bZIP-homolog genes is a conserved event in alcelaphine herpesvirus 1. *Sci. Rep.* **6**, 38607; doi: 10.1038/srep38607 (2016).

**Publisher's note:** Springer Nature remains neutral with regard to jurisdictional claims in published maps and institutional affiliations.

## Supplementary Material

Supplementary Figures

## Figures and Tables

**Table 1:** Sequence differences in LUR between strain C500 (GenBank accession AF005370) and the BAC clone.

Region	Location in reference (nt)	Location in BAC (nt)	Nucleotide change	Amino acid change
ORF18	35,830	45,916	A > G	Ile > Val
ORF25	35,830	57,915	C > A	Thr > Asn
ORF25	49,661	59,607	T > C	Met > Thr
A7	77,214	87,160	A > G	Thr > Ala
A8	79,330	89,276	C > T	Arg > Cys
Noncoding region (ORF53-ORF54)	80,619	90,575	T > C	Noncoding
Noncoding region (ORF69-ORF73)	111,028	120,995	T insertion	Noncoding
Noncoding region (ORF69-ORF73)	111,866	121,834	C > T	Noncoding
ORF73	119,851	130,297	C > A	Val > Gly
ORF73	119,932	129,825	C > A	Val > Gly
ORF73	119,950	129,851	72 bp insertion	Noncoding
Noncoding region (A9.5-A10)	128,305	138,422	52 bp deletion	Noncoding
Noncoding region (near right end)	130,562	140,604	A > T	Noncoding

**Table 2:** Oligonucleotides used in this study.

Sequence	Primer	Primer sequence (5′-3′)
β-lactamase gene	LUR-C500DT-AmpR-Fwd	GTCCTTTTCAATCATATTGTCTTCTGTTTGAATCAGGCTAACTAGCACCAGTGCGCGGAACCCCTATTTG
	LUR-C500DT-AmpR-Rev	GCTCTGGTGGGGAAAGGTTAGGTTGCCAATTGTTATATAAACCAATTGCTTTACCAATGCTTAATCAGTG
galK sequence	TR-C500DT-galK-Fwd	AAGAGGCCAGCACAGTAGCAGTCATCTGTTTTACAAAAAGTCCCCCTTAACCTGTTGACAATTAATCATCGGCA
	TR-C500DT-galK-Rev	TATAGAAAGCCAAGCAGCCCAGGGCAAAGGCAGCTATCATTAAATTTGTGTCAGCACTGTCCTGCTCCTT
TR-C500DT	C500DT-clean-Fwd	AGAGGCCAGCACAGTAGCAGTCATCTGTTTTACAAAAAGTCCCCCTTAACACAAATTTAATGATAGCTGCCTTTGCCCTGGGCTGCTTGGCTTTCTAT
	C500DT-clean-Rev	ATAGAAAGCCAAGCAGCCCAGGGCAAAGGCAGCTATCATTAAATTTGTGTTAAGGGGGACTTTTTGTAAAACAGATGACTGCTACTGTGCTGGCCTCT
galK sequence	LUR-C500DT-galK-Fwd	GTCCTTTTCAATCATATTGTCTTCTGTTTGAATCAGGCTAACTAGCACCACCTGTTGACAATTAATCATCGGCA
	LUR-C500DT-galK-Rev	GCTCTGGTGGGGAAAGGTTAGGTTGCCAATTGTTATATAAACCAATTGCTTCAGCACTGTCCTGCTCCTT
LUR-C500DT	LUR-C500DT-Fwd	GTCCTTTTCAATCATATTGTC
	LUR-C500DT-Rev	GCTCTGGTGGGGAAAGGTTAG
C500DT/left TR-LUR junction	A6-ATG-Fwd	ATGCATAAGCACTCTGCTACAGC
	Left-end-C500-Rev	GGCTATCTGCTGCGCGGCGC
