# Supplementary material for: Genomic duplication and translocation of reactivation transactivator and bZIP-homolog genes is a conserved event in alcelaphine herpesvirus 1
Source: Sci Rep. 2016 Dec 7;6:38607. doi: 10.1038/srep38607 (PMC5141506; doi:10.1038/srep38607)
Supplement: Supplementary Figures [file srep38607-s1.pdf]

for Scientific Reports

Supplementary material

Genomic duplication and translocation of reactivation transactivator and bZIP-homolog genes is a conserved event in alcelaphine herpesvirus 1

Françoise Myster[1,*], Steven J. van Beurden[1,*,¶], Océane Sorel[1,§], Nicolás M. Suárez[2], Alain Vanderplasschen[1], Andrew J. Davison[2], Benjamin G. Dewals[1]

[1]Fundamental and Applied Research in Animals and Health (FARAH), Immunology-Vaccinology, Faculty of Veterinary Medicine (B43b), University of Liège, Belgium, [2]MRC - University of Glasgow Centre for Virus Research, Sir Michael Stoker Building, Glasgow G61 1QH, UK

*Contributed equally to the study
[¶]Current address: Pathology Division, Department of Pathobiology, Faculty of Veterinary Medicine, Utrecht University, P.O. Box 80.158, 3508 TD, Utrecht, The Netherlands
[§]Current address: Department of Molecular Microbiology and Immunology, University of Southern California, Health Sciences Campus, CA 90033, Los Angeles, USA

***Correspondence**: Fundamental and Applied Research in Animals and Health (FARAH), Immunology-Vaccinology, Faculty of Veterinary Medicine (B43b), Quartier Vallée 2, Avenue de Cureghem 10, University of Liege, Liege, Belgium - Phone: 32-4-366 42 69 - Fax: 32-4-366 42 61 - E-mail: bgdewals@ulg.ac.be*

```
C500DT          ------------------------------------------------ccaagaggccag    12
WC11DT          tgtccttttcaatcatattgtcttctgtttgaatcaggctaactagcaccaagaggccag    60
                                                                ************

C500DT          cacagtagcagtcatctgttttacaaaaagtccccccttaaaaaattaaggcaggcttggg    72
WC11DT          cacagtagcagtcatctgttttacaaaaagtccccccttaaaaaattaaggcaggcttggg    120
                ************************************************************

C500DT          tatttttatgtgagctaaagcttgctatcagcaggcgccacttggtctgttggttagatg    132
WC11DT          tatttttatgtgagctaaagcttgctatcagcaggcgccacttggtctgttggttagatg    180
                ************************************************************

C500DT          taagaatagcaacaggaactggaagctccatggttgtgaatcaagttaaaa-aaaagtag    191
WC11DT          taagaatagcaacaggaactggaagctccatggttgtgaatcaagttaaaaaaaaagtag    240
                *************************************************** ********

C500DT          gtgacaggcttaaataacaagccacaggaactgtactcactaaatctgaccagattgttt    251
WC11DT          gtgacaggcttaaataacaagccacaggaactgtactcactaaatctgaccagattgttt    300
                ************************************************************

C500DT          aagcagccagtgagtaagctcacatttatttctctaaggcttcatttcacaggcacacaa    311
WC11DT          aagcagccagtgagtgagctcacatttatttctctaaggcttcatttcacaggcacacaa    360
                *************** ********************************************

C500DT          atttgactacccaaaatgagtgccaacaaccccttcatgtgcatccagagatcctcctcct    371
WC11DT          atttgactacccaaaatgagtgccaacaaccccgcatgtgcatccagagatcctcctcct    420
                ********************************* **************************

C500DT          aaaaaggtaaggactttttttttcttctatcaggtcatttttaaatggagacttatcttcc    431
WC11DT          aaaaaggtaaggactttttttttcttctatcaggtcatttttaaatggagacttatcttcc    480
                ************************************************************

C500DT          cgggaaacagtaaaccacatgtgttggacttaaagtttgcttgatctaattacttgatta    491
WC11DT          cgggaaacagtaaaccacatgtgttggacttaaagtttgcttgatctaattacttgatta    540
                ************************************************************

C500DT          tgtaacagccttttatactaacatatttatttttaggatgattaataataaacaatgact    551
WC11DT          tgtaacagccttttatactaacatatttatttttaggatgattaataataaacaatgact    600
                ************************************************************

C500DT          atcctaaaaatgtttttgcatactttaaggtttaaaaatctaaattatatttttttaaatc    611
WC11DT          atcctaaaaatgtttttgcatactttaaggtttaaaaatctaaattatatttttttaaatc    660
                ************************************************************

C500DT          atttacagaggagactatcaagaccaatatgcatagatgacttcatagacatcactgct    671
WC11DT          atttacagaggagactatcaagaccaatatgcatagatgacttcatagacatcactgct    720
                ************************************************************

C500DT          gatcttggtgatacaataggggctgctttaaagtccttccagcaaaataatgcttgcact    731
WC11DT          gatcttggtgatacaataggggctgctttaaagtccttccagcaaaataatgcttgcact    780
                ************************************************************

C500DT          caagaacagagtgagcagtttaccagggaagtatatgatgtctgcaaaaatattctccag    791
WC11DT          caagaacagagtgagcagtttaccagggaagtatatgatgtctgcaaaaatattctccag    840
                ************************************************************

C500DT          gagaacaaatttagaaatgaaatgtttggatttgttgctgatatgaacctgctaaatcta    851
WC11DT          gagaacaaatttagaaatgaaatgtttggatttgttgctgatatgaacctgctaaatcta    900
                ************************************************************

C500DT          tttgccctgttcagaagctataagcagagagttaggacccactttggaaaacagcttcta    911
WC11DT          tttgccctgttcagaagctataagcagagagttaggacccactttggaaaacagcttcta    960
                ************************************************************

C500DT          tgtgccactgcctcatctcaaataattagattctttttagaaagagttataaggcacact    971
WC11DT          tgtgccactgcctcatctcaaataattagattctttttagaaagagttataaggcacact    1020
                ************************************************************

C500DT          gacaaatggtttttgttagccccctgcaatggcctcattctcccacaagagctggcgaag    1031
WC11DT          gacaaatggtttttgttagccccctgcaatggcctcattctcccacaagagctggcgaag    1080
                ************************************************************

C500DT          gaaatgtatgttctcctgtccgaagccagggggaaagctttgaaccaagggagaatgttt    1091
WC11DT          gaaatgtatgttctcctgtccgaagccagggggaaagctttgaaccaagggagaatgttt    1140
                ************************************************************

C500DT          agcggagggagacaaaacatgatgaatgccgccaagaaggttttgacagtgtactctagc    1151
WC11DT          agcggagggagacaaaacatgatgaatgccgccaagaaggttttgacagtgtactctagc    1200
                ************************************************************

C500DT          ctcagagacgatggagaaatcagtccagaagtaaaggcttacatggcttacattttccca    1211
WC11DT          ctcagagacgatggagaaatcagtccagaagtaaaggcttacatggcttacattttccca    1260
                ************************************************************

C500DT          gtgcctgacattgagcaggtattccaaccactgtttaagttggagcaagaaatcagaaaa    1271
WC11DT          gtgcctgacattgagcaggtattccaaccactgtttaagttggagcaagaaatcagaaaa    1320
                ************************************************************

C500DT          ggtaaagccaccttgacacagtctctgctgtttgcaccaaggaaaagatgtcccaataag    1331
WC11DT          ggtaaagccaccttgacacagtctctgctgtttgcaccaaggaaaagatgtcccaataag    1380
                ************************************************************

C500DT          actgtgttctcacagtatggggccaggcagaggtatgtgttaccagaagttttactggaa    1391
WC11DT          actgtgttctcacagtatggggccaggcagaggtatgtgttaccagaagttttactggaa    1440
                ************************************************************

C500DT          gcaagtgaaccacagtcaaccctagcttacgggtgccctgacatttcatctcttttgaga    1451
WC11DT          gcaagtgaaccacagtcaaccctagcttacgggtgccctgacatttcatctcttttgaga    1500
                ************************************************************

C500DT          gattcatcttcaactcaagaaaatacagacgaagaatctggcccgtgctgcagcaagact    1511
WC11DT          gattcatcttcaactcaagaaaatacagacgaagaatctggcccgtgctgcagcaagact    1560
                ************************************************************

C500DT          ctcagcccaggtgtgccacagcctcaatctgaatatgaccccagcccgacctctccacct    1571
WC11DT          ctcagcccaggtgtgccacagcctcaatctgaatatgaccccagcccgacctctccacct    1620
                ************************************************************

C500DT          gattcggacactgagtcatgtgatagccaggttcgtccagaaagtactgactccgacacc    1631
WC11DT          gattcggacactgagtcatgtgatagccaggttcgtccagaaagtactgactccgacacc    1680
                ************************************************************

C500DT          catgcagaagacgatgatgttcctgaagcaccccaagcagcaagccaaactcagccaaca    1691
WC11DT          catgcagaagacgatgatgttcctgaagcaccccaagcagcaagccaaattcagccaaca    1740
                ************************************************* **********

C500DT          actacccaagagacacagagttgctgcagtgtctacaatccagctaccactcagacggga    1751
WC11DT          actacccaagagacacagagttgctgcagtgtctacaatccagctaccactcagacggga    1800
                ************************************************************

C500DT          ttctactataaccagcagtcaagtgataattttgcctctcgtatagatgcatctgccaca    1811
WC11DT          ttctactataaccagcagtcaagtgataattttgcctctcgtatagatgcatctgccaca    1860
                ************************************************************

C500DT          tcaaccagttatgggtatccaggcccagtcactaatcacggcttttcaacttctgtcttt    1871
WC11DT          tcaaccagttatgggtatccaggcccagtcactaatcacggcttttcaacttctgtcttt    1920
                ************************************************************

C500DT          tcccatacagtacccacaacggccactgggcagcagcagctgcatcaaaatatgtatggg    1931
WC11DT          tcccatacagtacccacaacggccactgggcagcagcagctgcatcaaaatatgtatggg    1980
                ************************************************************

C500DT          ggtggtcagcagacgaccacatacggcagctatgtgggggggttactcagatgctaatggc    1991
WC11DT          ggtggtcagcagacgaccacatacggcagctatgtgggggggttactcagatgctaatggc    2040
                ************************************************************

C500DT          cagtctgttggtgcaagtaccagttactacaccagtaagcctgcgaccagccgcagcagc    2051
WC11DT          cagtctgttggtgcaagtaccagttactacaccagtaagcctgcgaccagccgcagcagc    2100
                ************************************************************

C500DT          accgctctgcagtggaccactttgtttccaccggccagctcatctgcttccagcagccaa    2111
WC11DT          accgctctgcagtggaccactttgtttccaccggccagctcatctgcttccagcagccaa    2160
                ************************************************************

C500DT          gtttctgattatttcagcggcttcccttattttcctggcagttctacagttcctcaggcc    2171
WC11DT          gtttctgattatttcagcggcttcccttattttcctggcagttctacagttcctcaggcc    2220
                ************************************************************

C500DT          ttcgagcctttagcgccatctacccctagcttgctggatgaactgcttgatagagacagt    2231
WC11DT          ttcgagcctttagcgccatctacccctagcttgctggatgaactgcttgatagagacagt    2280
                ************************************************************

C500DT          ggacttgtaagccagcagcaggcacctgcacctccccaaaatgaccaggggggggcctcct    2291
WC11DT          ggacttgtaagccagcagcaggcacctgcacctccccaaaatgaccaggggggggcctcct    2340
                ************************************************************

C500DT          cagtatgtgcccgttgctcaagagcaacagcagtccagtacagacccgctgtctgatgag    2351
WC11DT          cagtatgtgcccgttgctcaagagcaacagcagtccagtacagacccgctgtctgatgag    2400
                ************************************************************

C500DT          atgaggagaatttttgagtttttttgacagtgtgaaccccgtgaccagaccatagccatat    2411
WC11DT          atgaggagaatttttgagtttttttgacagtgtgaaccccgtgaccagaccatagccatat    2460
                ************************************************************

C500DT          ttgggagtgtaaacagtaacctctgtaaactgccttttttatgtgaaatgcgtctctctg    2471
```

```
WC11DT          ttgggagtgtaaacagtaacctctgtaaactgcctttttatgtgaaatgcgtctctctg    2520
                ************************************************************

C500DT          ccaaaactatctgctctgttttgtacaaaaagtggaagtgtgtgtttatatgattcacaa    2531
WC11DT          ccaaaactatctgctctgttttgtacaaaaagtggaagtgtgtgtttatatgattcacaa    2580
                ************************************************************

C500DT          aactgtagtttaacagtaaaagttaattgtggtttgtggtatgacacacagttctggaaa    2591
WC11DT          aactgtagtttaacagtaaaagttaattgtggtttgtggtatgacacacagttctggaaa    2640
                ************************************************************

C500DT          ctgaccaaacagtgttcttatgtgcacttattttgtatttcccttatgcctgccagagtg    2651
WC11DT          ctgaccaaacagtgttcttatgtgcacttattttgtatttcccttatgcctgccagagtg    2700
                ************************************************************

C500DT          ctcaataaaagttacactcaagtgccactgagagtttttctttttcaacactataaaa    2711
WC11DT          ctcaataaaagttacactcaagtgccactgagagtttttctttttcaacactataaaa    2760
                ************************************************************

C500DT          gcatctttggggactcatgcataagcactctgctacagctgacatgctacccatttcagc    2771
WC11DT          gcatctttggggactcatgcataagcactctgctacagctgacatgctacccatttcagc    2820
                ************************************************************

C500DT          atttagccctctgaaattcttgggtggagtggtggctacaccccaggggccagtattgtg    2831
WC11DT          atttagccctctgaaattcttgggtggagtggtggctacaccccaggggccagtattgtg    2880
                ************************************************************

C500DT          ggatgccactactactcctgtccctttccaggctttcccctgccctccggcaataactcc    2891
WC11DT          ggatgccactactactcctgtccctttccaggctttcccctgccctccggcaataactcc    2940
                ************************************************************

C500DT          tataaatcagtttgttaattatcagcctgcgcctgcgatggataagatatggagacctgc    2951
WC11DT          tataaatcagtttgttaattatcagcctgcgcctgcgatggataagatatggagacctgc    3000
                ************************************************************

C500DT          ctttgaggactaccgtggggtaactatgagaccggccttaccggaggtacctgaaacaag    3011
WC11DT          ctttgaggactaccgtggggtaactatgagaccggccttaccggaggtacctgaaacaag    3060
                ************************************************************

C500DT          tttgagaccagctttacaggagctacccgagccatcatctcctcaaagtcagagttctgt    3071
WC11DT          tttgagaccagctttacaggagctacccgagccatcatctcctcaaagtcagagttctgt    3120
                ************************************************************

C500DT          ggatgatgacacagattctaaagaagatgttacagagaccttagaatgtgctcaggctct    3131
WC11DT          ggatgatgacacagattctaaagaagatgttacagagaccttagaatgtgctcaggctct    3180
                ************************************************************

C500DT          gacagatctgaagtggggtacagtggaccctccgagaagcacatctccagttactgcttc    3191
WC11DT          gacagatctgaagtggggtacagtggaccctccgagaagcacatctccagttactgcttc    3240
                ************************************************************

C500DT          tacttcaagcggcgtgtccagcgatttgcagaaggcaaaggaacaggctatacgccgcta    3251
WC11DT          tacttcaagcggcgtgtccagcgatttgcagaaggcaaaggaacaggctatacgccgcta    3300
                ************************************************************

C500DT          cagacttattgaagctaaagacttgggcaagctagtagtgagaggacccaaaaaagacaa    3311
WC11DT          cagacttattgaagctaaagacttgggcaagctagtagtgagaggacccaaaaaagacaa    3360
                ************************************************************

C500DT          gcgagatccagacttctacatcaaaaaggtacctttacattttcttaaaattaaaagta    3371
WC11DT          gcgagatccagacttctacatcaaaaaggtacctttacatttgtcttaaaattaaaagta    3420
                ****************************************** *****************

C500DT          actttttaggttgcatgaagctaacactttttttctctttttatagatacagcagagtac    3431
WC11DT          actttttaggttgcatgaagctaacactttttttctctttttatagatacagcagagtac    3480
                ************************************************************

C500DT          cacaaaggcagaggtgcgcagatggagtacaagattgcatgctaaactctttagagaaaa    3491
WC11DT          cacaaaggcagaggtgcgcagatggagtacaagattgcatgctaaactctttagagaaaa    3540
                ************************************************************

C500DT          ttaccacaaacgaatacttcagatgatgaaggtaaaggacttccccgtataaatataccc    3551
WC11DT          ttaccacaaacgaatacttcagatgatgaaggtaaaggacttccccgtataaatataccc    3600
                ************************************************************

C500DT          ccgtgccctttggggaaaagagtacctaaacacttaatactctttttttacactccaggag    3611
WC11DT          ccgtgccctttggggaaaagagtacctaaacacttaatactctttttttacactccaggag    3660
                ************************************************************

C500DT          ctggaagataaaacaaaagaactgaaatttttacaggagagtaccttgcggcttttaaaa    3671
WC11DT          ctggaagataaaacaaaagaactgaaatttttacaggagagtaccttgcggcttttaaaa    3720
                ************************************************************

C500DT          cagaatgagaaactaagggcacggctggcttccctgcatgtaaaaacatcagctacgaac    3731
WC11DT          cagaatgagaaactaagggcacggctggcttccctggaatgcaaacttgagtttagcatt    3780
                ************************************ *    ***        **  *

C500DT          taaacccccgccccattctaccctcaatctataaataaagttttgtttgcacccattttt    3791
WC11DT          tctcagctccaaaggtaaagagta--------cgccggcgttctgctc-caagcctttga    3831
                *     * *      *                       *** ** *  **  * ***

C500DT          atgtgtttgtgtgtat----------gtttaggggtgggtattggttttaaaaagtaaaa    3841
WC11DT          acctagtacaaggcctcctccaggtaccgtagctcccggtattttatcaac-tactgca-    3889
                *  *  *     *  *             ***     ******   *  *   * *  *

C500DT          gattagtattgtgacatctgggacaaagaaactattttaccttttcaggtatgagacttt    3901
WC11DT          ------aattttgaaaccagtactaataaaagtagccccacatacacccccacgccagct    3943
                       *** *** * * *    **  *** **      * *                *

C500DT          accctattgacacactcttaagtaactatttaagatattaatgttttgtaagccttgcct    3961
WC11DT          aaactctccacacccccaggattaactaa---------taccctct---------tgctc    3985
                *  ** *  **** * *   * ******          **   * *         ***

C500DT          acgtaccgggagtgtaaataactttaagagcttccccgtaact------------cgtat    4009
WC11DT          acagcaggagagcataact--caggcataggctccactttagagccatttacaacagtat    4043
                **     * ***  *** *  *    * **  *** * * *               ****

C500DT          gtattctgtttatttagtgtgactgtattttaaaaatatttaactgtggctgggctctga    4069
WC11DT          cagttcaagtacttcagacaccctcttcccctaccagggataccagtaccttggtaataa    4103
                   ***   *  ** **     ** *      *  *    ** * **  ** **   * *

C500DT          gatgttggcagaaatgttgtggcctgcagtgaacatgctgcttccccgtaaagcactttt    4129
WC11DT          aa-ctaaccaca------gt----------------tccccaagaccacaaagcagtgtc    4140
                 *  *   ** *      **                 *  *    **  ****** * *

C500DT          ggtagacattttttttattttggcagccacaaatttaatgatagctgcctttgccctggg    4189
WC11DT          ac---caagtttagta---acccccgggaggacttccactttg-----------------    4177
                       * ***  *        * *  *  * **  *   *

C500DT          ctgcttggctttctataag      4208
WC11DT          -------------------      4177
```

Supplementary Figure S1. Alignment of C500DT with WC11-DT using Clustal Omega (http://www.ebi.ac.uk/Tools/msa/clustalo/). Sequence highlighted in green indicates ORF50 exons. Sequence highlighted in pink indicates A6 coding sequence. Yellow highlights indicate single nucleotide polymorphism. Red sequence shows region of C500DT and WC11-DT with no sequence similarity.

```
WC11DT-end       ------------------------------------------------------------
C500-LUR         gcacggctggcttccctgcatgtaaaaacatcagctacgaactaaaccccgccccattc

WC11DT-end       ------------------------------------------------------------
C500-LUR         taccctcaatctataaataaagttttgtttgcacccatttttatgtgtttgtgtgtatgt

WC11DT-end       ------------------------------------------------------------
C500-LUR         ttaggggtgggtattggttttaaaaagtaaaagattagtattgtgacatctgggacaaag

WC11DT-end       ------------------------------------------------------------
C500-LUR         aaactattttaccttttcaggtatgagactttaccctattgacacactcttaagtaacta

WC11DT-end       ------------------------------------------------------------
C500-LUR         tttaagatattaatgttttgtaagccttgcctacgtaccgggagtgtaaataactttaag

WC11DT-end       ------------------------------------------------------------
C500-LUR         agcttccccgtaactcgtatgtattctgtttatttagtgtgactgtattttaaaaatatt

WC11DT-end       ------------------------------------------------------------
C500-LUR         taactgtggctgggctctgagatgttggcagaaatgttgtggcctgcagtgaacatgctg

WC11DT-end       ------------------------------------------------------------
C500-LUR         cttccccgtaaagcactttggtagacattttttttattttggcagccacaaatttaatg

WC11DT-end       ------------------------------------------------------------
C500-LUR         atagctgcctttgccctgggctgcttggctttctataagcaattggtttatataacaatt

WC11DT-end       ------------------------------------------------------------
C500-LUR         ggcaacctaacctttccccaccagagcggggatgaggttattagggccatgtacataccc

WC11DT-end       ------------------------------------------------------------
C500-LUR         ccagtcaatgacagtgtggattttaaccctgggttcagacttagctggttaaacacccta

WC11DT-end       ------------------------------------------------------------
C500-LUR         agtccgctctccgatgggccctatgactcttggtcccagtgcgagatctgccccggtcgc

WC11DT-end       ------------------------------------------------------------
C500-LUR         tttgttggccaaaaggcctgttattatgtccccccaaagacctatagctttcaaaactgc

WC11DT-end       ------------------------------------------------------------
C500-LUR         ttctttgcctgtaagaatatttctaaatgtttttatctttatactccacagaatataact

WC11DT-end       ------------------------------------------------------------
C500-LUR         gatccttttttttgaccacaccttacgagaccaggatatctggatagggactttttttaaa

WC11DT-end       ------------------------------------------------------------
C500-LUR         aagcttaatgctgccctgagcaccatagataataactttgactacacagcgtgggatgag

WC11DT-end       ------------------------------------------------------------
C500-LUR         cttagtgtttactgtgcctacctgacacgcaggtcccgatccacggtctattttacagac

WC11DT-end       ------------------------------------------------------------
C500-LUR         tgcactgctagcaagctctgtctgtgtggccaagaggattttaccccagcacccttctac

WC11DT-end       ------------------------------------------------------------
C500-LUR         gaggccctgaccccagcacccgtccatggataactatacactagccctaaactgcaacca

WC11DT-end       ------------------------------------------------------------
C500-LUR         caccacccacggctacgcgcactacgtctactccactctgagctacctggtgtttattgg

WC11DT-end       ------------------------------------------------------------
C500-LUR         aaatgcacctggatatccaaactgtgaaaactgtatctatgacataaccttcaacagcac

WC11DT-end       ------------------------------------------------------------
C500-LUR         ctctatgttgaatatcagcaacaattatttccatatagctaatgccaccctcggagcccc

WC11DT-end       ------------------------------------------------------------
C500-LUR         tgaatatccagaacagccagtaactctcagtgttataggagaggaaagcccctgggttgc

WC11DT-end       ------------------------------------------------------------
C500-LUR         ctggctcgcagtacatacgcctgacaaaaactacacagaagatgctaataccgctcgggc

WC11DT-end       ------------------------------------------------------------
C500-LUR         cgtgatgagggtcctagatccttttaacatatcttggtgtgccgtgtacaacgtgcagtc

WC11DT-end       ------------------------------------------------------------
C500-LUR         cgtggacccgacagacactcaagatattagtaactgcacagaagtttcagaagagctgcc

WC11DT-end       ------------------------------------------------------------
C500-LUR         agtgaacaatcagaagtcctcaattcatctcaacacctactttttaaactctacctttac

WC11DT-end       ------------------------------------------------------------
C500-LUR         tgtttctgtatccctaaacgcctctaacatttctagtcagtgtagtggtaatgtgtccct

WC11DT-end       ------------------------------------------------------------
C500-LUR         agaaaatatgaccagtaatgtccatataccctgtcccaccctcccccctgactgtgagcgt

WC11DT-end       ------------------------------------------------------------
C500-LUR         caaggcctctacagacaatatgcagaccggggcagcggttaatgctagcatagacatagg

WC11DT-end       ------------------------------------------------------------
C500-LUR         ctggctcttgcaaaatctaactgacgcttccctgacagtcagttcatccccgggtaacct

WC11DT-end       ------------------------------------------------------------
C500-LUR         gacccaaaaaaactgcaccacttacaaaaatatttccaagacagacagacacgtacgaga

WC11DT-end       ------------------------------------------------------------
C500-LUR         cgagaacttgtacgtgctggctcaaattcccgctctagaaggccacaaggattccttcac

WC11DT-end       ------------------------------------------------------------
C500-LUR         gctaacgcggagtcctatcctgctgaactttagcagaaactctagcgagtttcagctagt

WC11DT-end       ------------------------------------------------------------
C500-LUR         gttgtatgataggaatagttcctgcgtgtgggccaacccggccaatgggaccgacacgat

WC11DT-end       ------------------------------------------------------------
C500-LUR         attggtctccatgtcctgtccccacatcacagccacaatctcaccaagaggtgagattaa

WC11DT-end       ---------------------------gaatgcaaacttgagtttagcatttctcagctc
C500-LUR         ggttaatgtgacagggaatttttcaaggaatgcaaacttgagtttagcatttctcagctc
                                            *********************************

WC11DT-end       caaaggtaaagagtacgccggcgttctgctccaagcctttgaacctagtacaaggcctcc
C500-LUR         caaaggtaaagagtacgccggcgttctgctccaagcctttgaacctagtacaaggcctcc
                 ************************************************************
```

```
WC11DT-end        tccaggtaccgtagctcccggtattttatcaactactgcaaattttgaaaccagtactaa
C500-LUR          tccgggtaccgtagctcccggtattttatcaactactgcaaattttgaaaccagtactaa
                  ***.********************************************************
WC11DT-end        taaaagtagccccacatacacccccacgccagctaaactctccacacccccaggattaac
C500-LUR          taaaagtagccccacatacacccccacgccagctaaactctccacacccccaggattaac
                  ************************************************************
WC11DT-end        taatacccctcttgctcacagcaggagagcataactcaggcataggctccactttagagcc
C500-LUR          taatacccctcttgctcacagcaggagagcataactcaggcataggctccactttagagcc
                  ************************************************************
WC11DT-end        atttacaacagtatcagttcaagtacttcagacaccctcttcccctaccagggataccag
C500-LUR          acttacaacagtatcagttcaagtacttcagacaccctcttcccctaccagggataccag
                  * **********************************************************
WC11DT-end        taccttggtaataaaactaaccacagttccccaagaccacaaagcagtgtcaccaagttt
C500-LUR          taccttggtaataaaactaaccacagttccccaagaccacaaaacagtgtcaccaagttt
                  *******************************************.****************
WC11DT-end        agtaacccccgggaggacttccactttg--------------------------------
C500-LUR          agtaacccccgggaggacttccactttgcccattgttagcatgactcacttttccaggga
                  ****************************
WC11DT-end        ------------------------------------------------------------
C500-LUR          aggttcgtcccccaaaccacaaactactgcagctaaaacatcctcagaggcttctttgcc

WC11DT-end        ------------------------------------------------------------
C500-LUR          acctttactcaccacaacccccaccccaactaacactgaaaaatctcagtcaacttttgc

WC11DT-end        ------------------------------------------------------------
C500-LUR          ttcttccactgtatcagtggatactacttttactggggatgatgtcaacaccgtggggac

WC11DT-end        ------------------------------------------------------------
C500-LUR          aatgtctcccagtataactcaaacactacccattactcctacatcaggtaggcagtacat

WC11DT-end        ------------------------------------------------------------
C500-LUR          agtagttggttgctgtaccctaaacagaaggtcaggtaacttatttttctttttttctttt

WC11DT-end        ------------------------------------------------------------
C500-LUR          tgttgcagcacatcgtgagagtgggcacaacacttcaatgcccaaccctcaccataattc

WC11DT-end        ------------------------------------------------------------
C500-LUR          tgttaagccagaagaccatccacaccacccagaaggagaccacccagacgcagaccacca

WC11DT-end        ------------------------------------------------------------
C500-LUR          tgagaggttccaaatttggctgctgcccatagcaggaacaatttttgcactagtggccct

WC11DT-end        -------------------------------------
C500-LUR          agttatagttaacatagctctatgtatgacagaataa
```

Supplementary Figure S2. Alignment of WC11DT region showing no sequence similarity with C500DT (Supplementary Fig. S1) and downstream sequence of strain C500 LUR sequence using Clustal Omega (http://www.ebi.ac.uk/Tools/msa/clustalo/) (coordinates of the LUR : nt 76,188 to nt 79,344 of refseq NC_002531.1). Sequence in bold indicates sequence present in C500-DT but not in WC11-DT. Sequence highlighted in blue shows A7 coding sequence of strain C500. Italicised sequence indicates A8 coding sequence of strain C500. Yellow highlights indicate single nucleotide polymorphism.
